# Comments on “Stunting is not a synonym of malnutrition”

**DOI:** 10.1038/s41430-020-0570-2

**Published:** 2020-01-27

**Authors:** Conny Tanjung, Titis Prawitasari, Damayanti Rusli Sjarif

**Affiliations:** 1 Nutrition and Metabolic Diseases Working Group, Indonesian Pediatric Society, Jakarta, Indonesia; 2 Pantai Indah Kapuk Hospital, Jakarta, Indonesia; 3 Department of Pediatrics Faculty of Medicine Universitas Indonesia, Dr Ciptomangunkusumo National Referral Hospital Jakarta, Jakarta, Indonesia

**Keywords:** Malnutrition, Paediatrics

## To the Editor:

After reading this paper published online on 29th of May 2019, we would like to raise our concern as it will mislead readers when they want to know about stunting. Stunting is a continuation process that does not happen abruptly. It starts with linear growth faltering process, where a child does not grow in length or height in accordance with his/her potential. It may begin very early in life, typically in utero, and generally continues during the first 2 postnatal years [1]. There are many important factors that can cause stunting such as household and family factors, inadequate infant-feeding practices and infection [1, 2]. The study by Scheffler et al. examined stunted children in 6–13.2-year-old age group, without trying to look for the causes of this condition. Stunted children can be caused by other factors such as familial short stature, constitutional delay of growth and development, genetic disorders, endocrine disorders, etc. Doing a study about stunting without identifying all these important factors might mislead the conclusion drawn from the study.

Scheffler et al. stated that the analysis of their study rejected the hypotheses all of which are based on the conventional definition of height stunting as due primarily to nutritional inadequacy [1]: stunted children are not uniformly characterized by depleted fat stores [2]; fat stores of less stunted children are not less depleted and better parental education does not minimize the risk of child undernutrition [3]; stunted children do not exhibit visible clinical signs of PEM. In their study they examined the 6–13.2-year-old children that were stunted. As the stunted growth might begin earlier, they might not find PEM signs and infections no more, moreover if they looked for these signs where actually the stunted children were not caused by stunting. As the children were recovered from stunting, they can have good nutritional status which are described as normal skinfold thickness/normal BMI and also can be found in overweight or obese because of the metabolic syndrome [3, 4]. The study from Rolfe et al. found that subjects who experienced early stunting had accumulated less fat-free mass that predisposed them towards obesity [3]. Study from Vonaesch et al. [4] found that stunting was associated with being overweight after some years with the adjusted OR of 3.21 (95% CI: 1.50; 6.90). So it is not mandatory to find the stunted children (caused by stunting process) have low skinfold thickness.

At the end of their study, Scheffler et al. questioned about the inappropriate use of global growth standards to conclude stunting and stimulated a debate about the inappropriate misapplication of a global growth reference derived from high socioeconomic and mostly westernized populations. Looking back to the history of how the WHO child growth standards was made, we knew it was based on longitudinal observation from five continents where children of well-off populations in developing countries experience similar growth patterns to those of healthy, well-nourished children in developed countries. A critical result of the study was the remarkable similarity in linear growth of the six Multicentre Growth Reference Study Group populations (3 and 70% interindividual and intersite variability, respectively), demonstrating that, when health, environmental, and care needs are met, the potential of growth is universal to at least 5 years of age. This growth standard is a symbol of children’s right to achieve their genetic growth potential [1].

Based on these available data, the conclusion of this paper should be questioned. The scientific evidence is clear and convincing that stunting is the result of poor nutrition, infection, together with socioeconomic influences and it is also clear that stunting is not synonymous with stunted.
